# A synergistic antioxidant nanozyme platform for corneal neovascularization treatment

**DOI:** 10.1039/d6na00297h

**Published:** 2026-08-14

**Authors:** Min Su, Tingxiu Ye, Wei Yuan, Bochao Zhang, Jinfa Ye, Chuan Chen, Chengchao Chu

**Affiliations:** a School of Pharmacy, Xiamen Medical College Xiamen 361023 China cc@xmmc.edu.cn; b Xiamen University Affiliated Xiamen Eye Center, Eye Institute of Xiamen University, School of Medicine, Xiamen University Xiamen 361102 China chuchengchao@xmu.edu.cn 787732914@qq.com

## Abstract

Corneal neovascularization (CNV) is recognized as one of the most common blinding eye diseases, afflicting millions of individuals globally each year. Substantial evidence has demonstrated that oxidative stress and inflammation constitute pivotal pathogenic factors in the initiation and progression of CNV. In this study, we prepared a synergistic nanozyme platform for the antioxidant therapy of CNV. Specifically, we co-assembled manganese(iii) *meso*-tetra(4-carboxyphenyl)porphine (Mn-TCPP) with octahydrated zirconium(iv) chloride, followed by surface modification with platinum nanoparticles (Pt NPs), preparing the Pt NPs-Mn-TCPP@Zr (PMTZ) nanomedicine. The prepared PMTZ nanomedicine showed enhanced antioxidant performance, which could inhibit the migration of human umbilical vein epithelial cells and eliminate excessive reactive oxygen species produced by oxidative stress cells. Finally, PMTZ was applied in the treatment of CNV, and its therapeutic efficacy was confirmed through experimental validation.

## Abstract 2

A synergistic antioxidant platform PMTZ was constructed *via* Mn-TCPP and Zr co-assembly with Pt nanoparticle modification. The PMTZ was applied for corneal neovascularization (CNV) treatment, and its therapeutic efficacy was fully validated.

## Graphical abstract

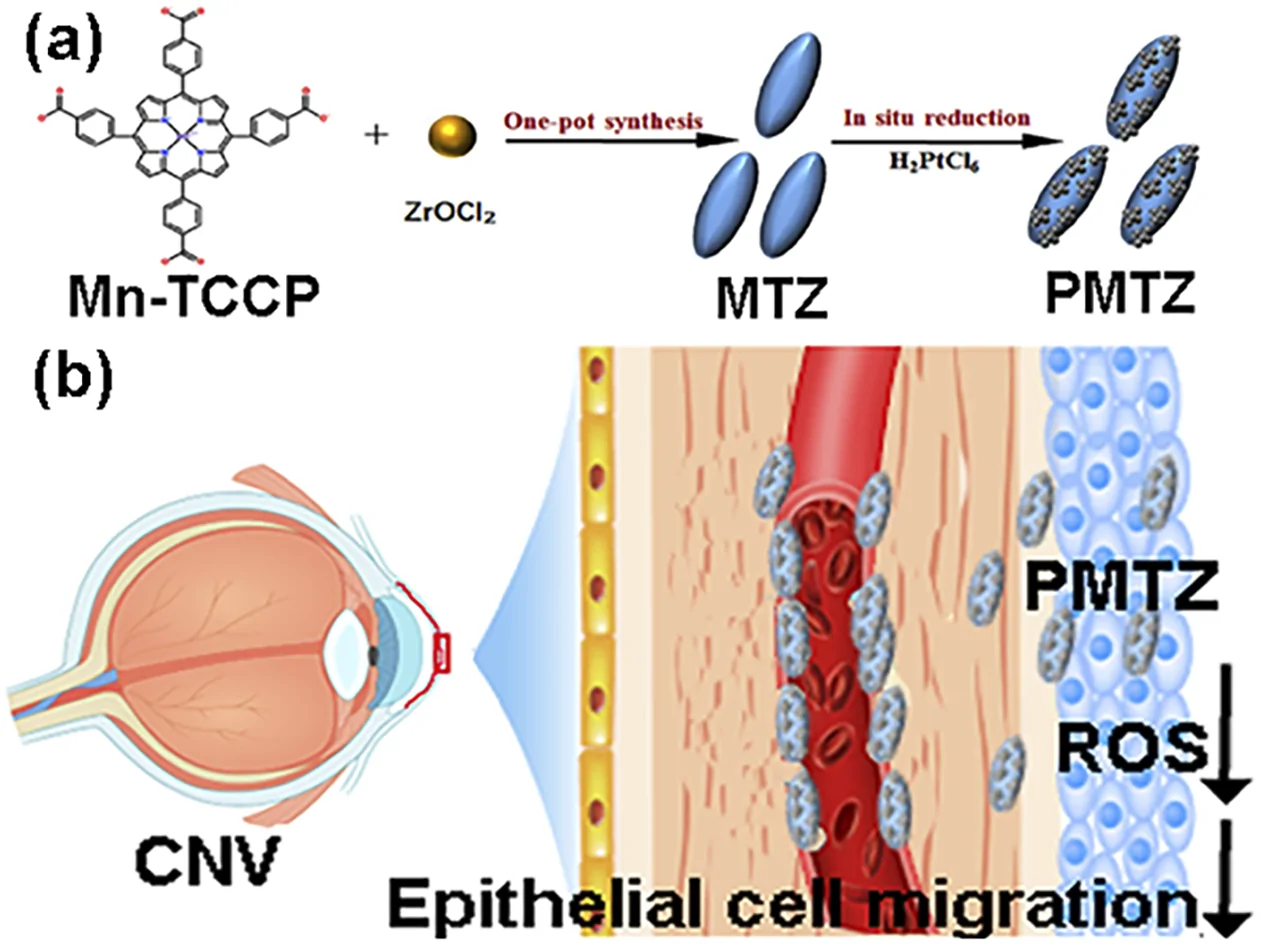

## 1 Introduction

Corneal neovascularization (CNV), affecting more than one million people each year globally, carries a risk of vision loss in approximately 12% of cases.^1^ Under physiological conditions, the cornea maintains a state of avascular transparency, a structural prerequisite for its critical refractive function.^2^ However, factors such as trauma, inflammation, and autoimmune diseases can affect the ocular area, causing vascular endothelial cells within the capillaries of the corneal margin to proliferate and migrate to the corneal stroma, resulting in CNV.^3–5^ In the treatment of CNV, topical steroids and nonsteroidal anti-inflammatory drugs remain the first-line choice.^6^ Two significant challenges persist in CNV treatment: poor corneal permeability of drugs like anti-VEGF agents and the adverse side effects linked to corticosteroid use.^7^

A series of studies have reported that oxidative stress and inflammation also play crucial roles in the occurrence and development of CNV.^1^ Excessive reactive oxygen species (ROS) in the CNV lesion area trigger multiple inflammatory responses: they disrupt redox-dependent signaling pathways that activate NF-κB in the cornea, cause oxidative damage to the extracellular matrix, induce endothelial cell apoptosis, and promote neovascularization, thereby exacerbating the inflammatory response.^8–10^ Therefore, excessive ROS is a key molecular driver in the onset and progression of CNV, making the restoration of ROS homeostasis a promising therapeutic strategy.^11–13^

Recently, biocompatible nanozymes that can mimic natural superoxide dismutase (SOD) and catalase (CAT), which can effectively eliminate ROS, have been used in CNV therapy.^14,15^ Wang *et al.*^16^ developed an active pharmaceutical ingredient (API) nanozyme (PC-DS NE), which effectively inhibited CNV in the corneal alkali burn model by eliminating ROS, inhibiting the expression of inflammatory factors and pro-angiogenic factors, and down-regulating ferroptosis. Cai *et al.*^17^ combined the nanozyme properties of vanadium carbide quantum dots (V_2_C QDs) with IL-10 gene therapy to construct a biomimetic metal–organic framework nano-drug MVPC, achieving a synergistic treatment of antioxidant and anti-inflammatory effects in CNV model rats. As a highly effective SOD mimic, manganese porphyrin (Mn-porphyrin) exerts potent antioxidant activity by catalytically scavenging superoxide anions, mitigating oxidative stress-induced cellular damage, and maintaining redox homeostasis in biological systems.^18–20^ Moreover, the use of Mn-porphyrin as a ligand in metal–organic framework (MOF) synthesis enhances the antioxidant catalytic performance of porphyrin-based systems by stabilizing the active Mn center, facilitating substrate accessibility, and enabling synergistic interactions between the porphyrin moiety and the framework structure.^3,21^ In addition, platinum nanoparticles (Pt NPs) anchored onto the surface of an antioxidant MOF could synergistically amplify its radical-scavenging capacity, significantly enhancing overall antioxidant performance through the combined effects of the MOF's inherent porous structure and platinum's catalytic activity.^22–24^

Hereby, we engineered biomimetic nanozyme Mn-TCPP@Zr (MTZ) nanoparticles by a one-step self-assembly procedure using Zr(iv) clusters and manganese *meso*-tetrakis(4-carboxyphenyl)porphyrin (Mn-TCPP). Subsequently, Pt NPs were anchored onto the surface of MTZ nanoparticles through an *in situ* growth strategy to construct the functional Pt NPs-Mn-TCPP@Zr (PMTZ) nanocomposite. The PMTZ nanozyme possessed stronger ROS-scavenging activity analogous to native SOD, while circumventing the issues of easy inactivation and rapid clearance associated with natural enzymes. Finally, PMTZ showed significant therapeutic efficacy in the CNV treatment, owing to their ability to effectively down-regulate ROS levels and suppress pathological angiogenesis.

## 2 Experimental

### 2.1 Synthesis of PMTZ nanoparticles

The MTZ nanoparticles were firstly constructed with 2,2′-bipyridine-5,5′-dicarboxylic acid (BPY-COOH) as the organic linker. In a typical synthesis, 427 mg of benzoic acid, 39 mg of zirconium oxychloride octahydrate (ZrOCl_2_·8H_2_O), 5 mg of Mn-TCPP, 2.9 mg of Bpy-COOH, and 75 mg of polyvinylpyrrolidone (PVP) were dissolved in 10 mL of *N*,*N*-dimethylformamide (DMF). The mixture was uniformly dispersed *via* ultrasonic treatment, then transferred to a 25 mL glass reactor and heated at 90 °C under continuous stirring (300 rpm) for 5 hours to facilitate MOF formation. Afterwards, the MTZ nanoparticles were collected using a centrifuge (14 000 rpm, 15 min), and then washed 3 times with 10 mL DMF.

### 2.2 Synthesis of PMTZ nanoparticles

The obtained MTZ intermediate (30 mg) was first dispersed into 10 mL of 3 mM chloroplatinic acid (H_2_PtCl_6_) aqueous solution. To ensure uniform adsorption of platinum precursor ions onto the MTZ surface, the mixture was magnetically stirred at 300 rpm for 12 h under ambient temperature conditions. Subsequently, 10 mL of 10 mM ascorbic acid solution was introduced as a mild reducing agent, and the reduction reaction was conducted at 35 °C with continuous stirring (300 rpm) for 5 h. This controlled reaction process facilitated the *in situ* nucleation and growth of platinum nanoparticles (Pt NPs) on the MTZ support, forming a stable PMTZ hybrid structure. Afterwards, the MTZ nanoparticles were collected using a centrifuge (14 000 rpm, 15 min), and then washed thrice with 10 mL ultrapure water. After the final wash, the purified PMTZ composite was redispersed in ultrapure water *via* ultrasonic treatment to achieve uniform colloidal dispersion. The resulting suspension was stored at 4 °C in a refrigerator for subsequent characterization and catalytic performance evaluation.

The morphology of the nanoparticles was studied by transmission electron microscopy (TEM) (DRTEM; JEOL, Tokyo, Japan), while the size distribution and zeta potential of nanoparticles (NPs) were measured using a Zetasizer (Malvern, UK).

### 2.3 *In vitro* cell studies

In subsequent investigations, human umbilical vein endothelial cells (HUVECs), obtained from Shanghai Zhong Qiao Xin Zhou Biotechnology Co., Ltd (product no. PRI-H-00023), were selected for *in vitro* cellular studies. First, PMTZ and MTZ were labeled with indocyanine green (ICG) *via* electrostatic adsorption to evaluate cellular uptake. HUVECs were seeded into glass-bottomed culture dishes at a density of 5 × 10^5^ cells per dish and cultured for 12 h. Subsequently, 20 µg mL^−1^ of ICG-labeled PMTZ or MTZ was added to the culture medium and incubated for 12 h. After incubation, the cells were washed with PBS, and nuclei were stained with 4′,6-diamidino-2-phenylindole (DAPI). Finally, cells were fixed with 4% paraformaldehyde, and fluorescence imaging was performed using a confocal laser scanning microscope (Zeiss LSM 880).

Subsequently, the *in vitro* ROS scavenging activity of PMTZ was studied. HUVECs were seeded into glass-bottomed culture dishes at a density of 5 × 10^5^ cells per dish for 12 h, and then H_2_O_2_ was added into the cells to enable the oxidative stress environment. After treating with PMTZ and MTZ for 12 h, cells were gently washed three times with PBS. To quantify intracellular ROS levels, cells were incubated with 10 µM 2′,7′-dichlorofluorescein diacetate (DCFH-DA) at 37 °C for 20 min in the dark. After probe loading, cells were washed three times with PBS to eliminate unincorporated DCFH-DA. Finally, fluorescence images were immediately acquired using a confocal laser scanning microscope.

The migratory capacity of HUVECs treated with PMTZ or MTZ was assessed using an *in vitro* scratch wound healing assay. HUVECs were seeded into 12-well plates at a density sufficient to reach 90–100% confluence within 24 h. Once confluent, a sterile 200-µL pipette tip was used to create a uniform linear scratch across the center of each well, mimicking a wound. Immediately after scratching, non-adherent cells and debris were gently removed by washing the wells twice with PBS. The culture medium was replaced with fresh serum-free medium containing PMTZ, MTZ, or untreated (control group), followed by incubation at 37 °C in a humidified incubator with 5% CO_2_. Bright-field images of the scratch area were captured using a Nikon Ti–U inverted fluorescence microscope at 0 h (immediately after treatment initiation) and 6, 12, and 24 h post-incubation. The relative migration rate was subsequently quantified using ImageJ software.

Total RNA was extracted from neovascularized corneas using Trizol Reagent (15,596,018; Invitrogen), the equal amount of RNA was reverse transcribed to cDNA using a HiScriptIVAll-in-One Ultra RT SuperMix for qPCR (R433-01, Vazyme); quantitative Real-Time PCR (qRT-PCR) was performed with a StepOneTM Real-Time PCR detection system (Applied Biosystems, Alameda, CA, USA) using a ChamQ Universal SYBR qPCR Master Mix (Q711-02, Vazyme).

### 2.4 *In vivo* CNV treatment of PMTZ

For *in vivo* study, male Sprague Dawley rats were procured from Shanghai SLAC Laboratory Animal Co., Ltd. All experimental procedures involving animals were reviewed and approved by the Animal Care and Use Committee (CC/ACUCC) of Xiamen University, with the ethics approval number XMULAC202220256. To establish the CNV model, ∼200 g rats were employed in this study. And a standard suture-induced method was employed: an 8–0 nylon thread was placed in the central corneal area of the rats' left eyes to trigger corneal neovascularization, which served as the basis for subsequent *in vivo* efficacy evaluation.

Following the successful establishment of the corneal neovascularization (CNV) animal model, *in vivo* drug enrichment was investigated. Male Sprague Dawley rats were randomly divided into two groups (*n* = 3 per group): the PMTZ group and the MTZ group, with both formulations labeled with ICG. A dose of 10 µL of 200 µg mL^−1^ PMTZ-ICG or MTZ-ICG was topically administered to the neovascularized left eyes of rats as eye drops. To assess the *in vivo* accumulation of the delivery systems, a Vevo 3100 photoacoustic imaging system (VisualSonics FujiFilm) was utilized. Specifically, photoacoustic imaging of oxygen saturation (PAI-o.s.) and ICG-mediated photoacoustic imaging (PAI-ICG) were performed on the neovascularized corneas at 3, 6, 12, and 24 hours post-administration. The obtained images were analyzed using the system's built-in software to quantify the photoacoustic signal intensity, which was correlated with the local drug concentration in the corneal tissue over time.

Additionally, we evaluated the *in vivo* therapeutic efficacy of the prepared antioxidant nanozyme. The model rats were randomly divided into three groups (*n* = 5 per group): the PMTZ group, MTZ group, and PBS (set as the control group). A 10 µL aliquot of 200 µg mL^−1^ PMTZ, MTZ, or PBS was topically administered to the neovascularized left eyes twice daily as eye drops. Slit lamp microscopy was performed daily (on days 1, 2, 3, 4, and 5 post-treatment initiation) to monitor the progression of corneal neovascularization in each group. At the end of the 5-day treatment period, all rats were euthanized, and the neovascularized corneas were harvested for CD31 immunohistochemical staining to assess histopathological changes and therapeutic outcomes at the tissue level.

Finally, isolated neovascularized corneas were extracted in a cold lysis buffer composed of protease and phosphatase inhibitors, and prepared for western blot (WB) assays with primary antibodies for Anti-NRF2(ab62352, Abcam, Cambridge, UK) and anti-VEGFA (66828-1-Ig, Proteintech). Actin (20536-1-AP, Proteintech) was used as the internal reference protein. These results were detected using an enhanced chemiluminescence reagent (E411-05; Vazyme) and recorded using a transilluminator (ChemiDoc XRS System; Bio-Rad, Philadelphia, PA, USA).

## 3 Results and discussion

**Fig. 1:**
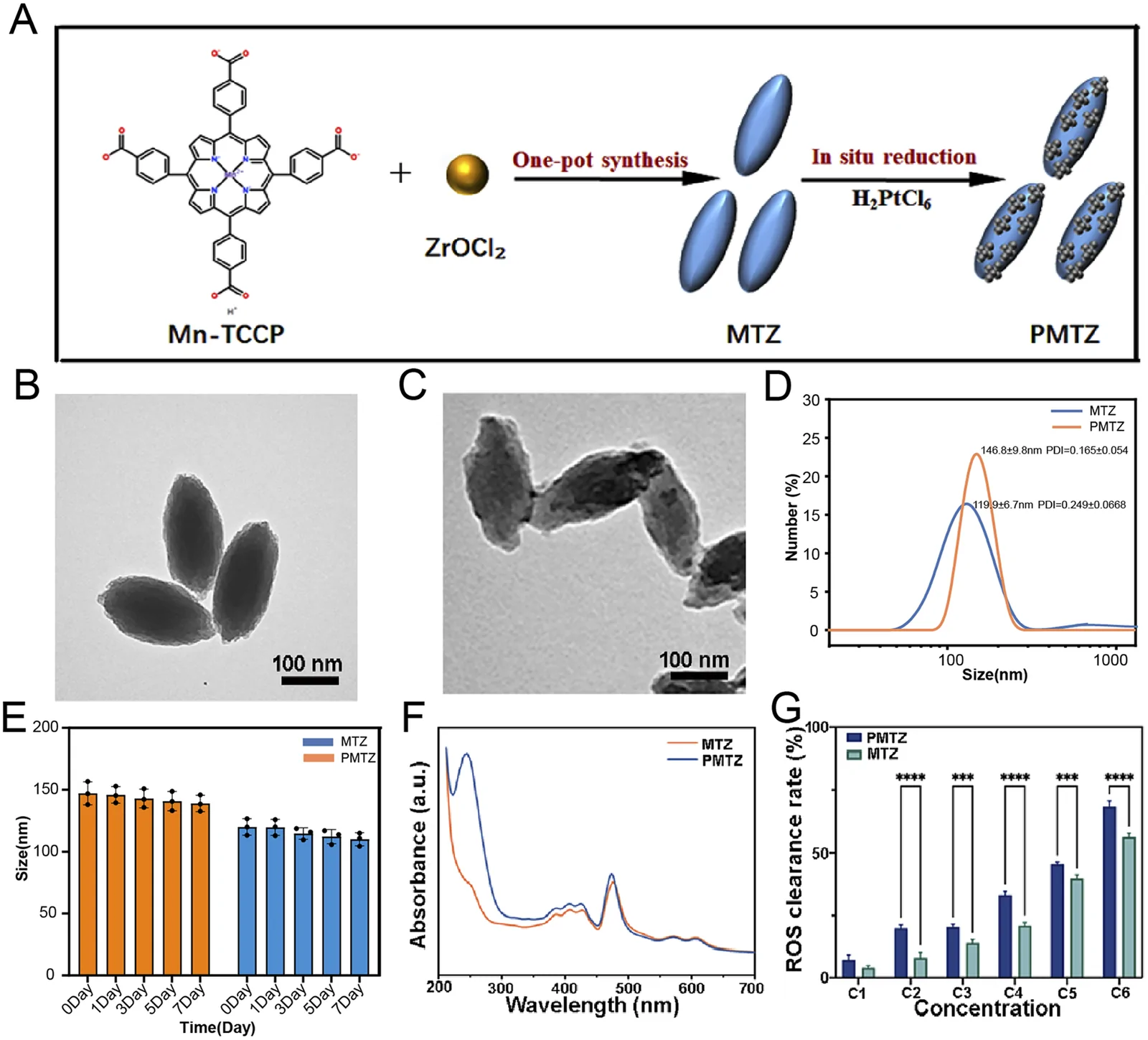
(A) Schematic illustration of PMTZ preparation. TEM images of MTZ (B) and PMTZ (C). (D) DLS of MTZ and PMTZ. (E) The stability of MTZ and PMTZ under physiological conditions. (F) UV-vis spectra of MTZ and PMTZ. (G) ROS scavenging rates of MTZ and PMTZ.

In this study, we utilized Zr ions, Mn-TCCP, and Bpy-COOH to assemble an antioxidant MTZ (Fig. 1A). The MTZ we synthesized exhibits a spindle-shaped nanostructure with a length of approximately 200 nm (Fig. 1B), demonstrating uniform dispersion and structural stability. Moreover, Pt NPs were grown on its surface *via* the reduction of platinum(iv) ions (Pt^4+^) using ascorbic acid as the reducing agent, with hexachloroplatinic acid (H_2_PtCl_6_) serving as the platinum precursor. As shown in Fig. 1C, the final product PMTZ also has a similar rough surface to MTZ, and the Pt NPs were modified on the surface of PMTZ. Owing to the attachment of Pt nanoparticles (Pt NPs) on both the exterior and interior structures, Pt NPs are visibly present at specific sites, offering enhanced catalytic active centers. Dynamic light scattering (DLS) was used to analyze the hydrodynamic size and stability of nanoparticles. As shown in Fig. 1D, the particle size of PMTZ increased by approximately 20 nm compared to MTZ, which is attributed to the deposition of platinum nanoparticles on the surface of the nanozyme. To further verify the stability of the nanozyme under physiological conditions, the nanozyme was dispersed in PBS solution containing 10% fetal bovine serum (FBS), and its particle size was analyzed at different time points. The results showed that the particle size of the nanozyme remained nearly unchanged after one week (Fig. 1E). In addition, the prepared PMTZ was characterized using ultraviolet-visible (UV-vis) spectroscopy (Fig. 1D). Within the wavelength range of 300–600 nm, PMTZ showed no distinct characteristic absorption peaks beyond those of MTZ. However, PMTZ exhibited a strong characteristic absorption band at approximately 255 nm, which can be attributed to the presence of surface-modified Pt NPs. Meanwhile, PMTZ exhibited a weak negative charge with a zeta potential of −5.3 mV, in distinct contrast to the positively charged MTZ, which had a zeta potential of 14.5 mV. The superoxide dismutase (SOD) enzyme activity was investigated to evaluate the antioxidant capacity of PMTZ and MTZ nanoparticles. As shown in Fig. 1E, both PMTZ and MTZ exhibited potent ROS-scavenging effects, with efficacy varying in a concentration-dependent manner. Notably, after loading with Pt nanoparticles, the ROS-scavenging ability of PMTZ was significantly enhanced compared to that of MTZ. This superior performance of PMTZ lays a solid foundation for its subsequent application in CNV antioxidant treatment.

**Fig. 2:**
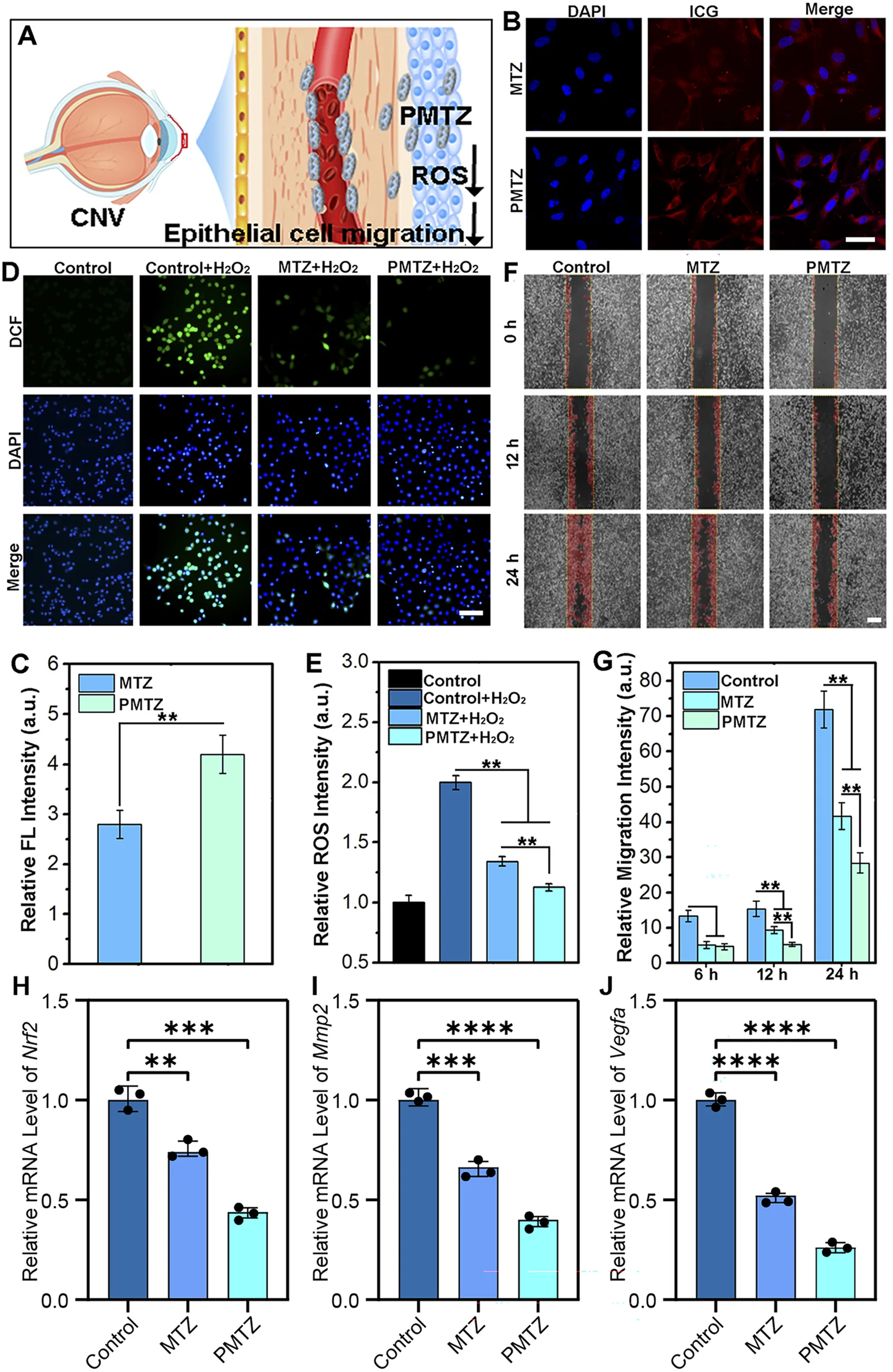
(A) Schematic illustration of the antioxidant therapy of PMTZ. Cell fluorescence images (B) and the quantitative fluorescence intensity (C) of MTZ and PMTZ. Cell ROS images (D) and the quantitative fluorescence intensity (E) of control, control + H_2_O_2_, MTZ + H_2_O_2_ and PMTZ + H_2_O_2_. Cell migration images (F) and relative migration rate (G) of HUVECs after treating with MTZ and PMTZ. The relative mRNA levels of NRF2 (H), MMP2 (I) and VEGFA (J) in HUVECs after treatment with MTZ and PMTZ.

In subsequent investigations, *in vitro* cellular studies of PMTZ were conducted using HUVECs (Fig. 2A). Briefly, HUVECs were incubated with ICG-labeled MTZ and PMTZ for 12 h, followed by observation under a laser scanning confocal fluorescence microscope. As shown in Fig. 2B and C, both MTZ and PMTZ effectively enriched into the cells, with PMTZ exhibiting higher cellular uptake efficiency than MTZ. This difference might be attributed to the weak electrical properties of PMTZ. The *in vitro* ROS scavenging activity of PMT in HUVECs was evaluated to assess its antioxidant potential under physiological conditions. Compared with the control group, PMTZ significantly reduced intracellular ROS levels (Fig. 2D and E), demonstrating its therapeutic potential for oxidative stress-related interventions. Given that ROS serve as critical signaling molecules in activating intracellular pathways that promote endothelial cell migration, the effect of PMTZ on HUVEC migration was subsequently investigated. As anticipated, the PMTZ treated group significantly inhibited cell migration compared to the other groups (Fig. 2F and G). The RNA was collected from each group for further analysis. The qRTPCR revealed that mRNA expression of NRF2, MMP-2 and VEGFA was significantly decreased in the PMTZ (Fig. 2H–J). The above results indicate that PMTZ can significantly inhibit angiogenesis *in vitro*.

**Fig. 3:**
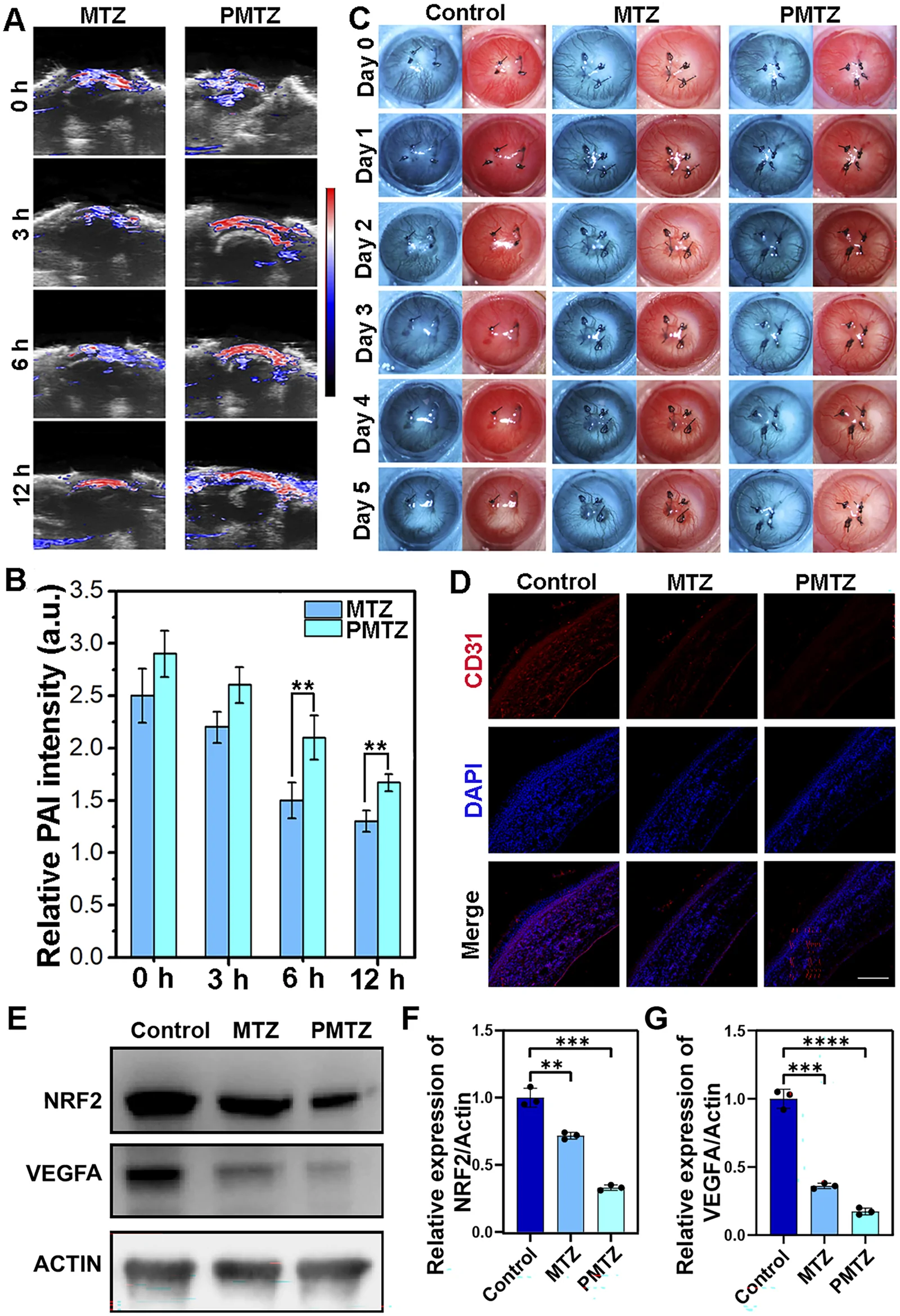
PAI (A) of the relative PA intensity (B) of cornea for MTZ and PMTZ treated group. *n* = 3. (C) Slit-lamp examination of CNV rat models at different time points following various treatments. (D) CD31 staining images of the cornea following different treatments. Scale bar = 200 µm. (E) Western blotting analysis of NRF2 and VEGFA expressing level in HUVECs after treating with control, MTZ and PMTZ. The gray-scale analysis of WB shows a decrease of NRF2 (F) and VEGFA (G) expression level in HUVECs after treating with control, MTZ and PMTZ.

To investigate the *in vivo* efficacy of PMTZ, we established a CNV rat model *via* a standard suture induction method. First, we conducted a drug accumulation study using photoacoustic imaging (PAI) with ICG-labeled PMTZ and MTZ. As depicted in Fig. 3A and B, both PMTZ and MTZ produced distinct photoacoustic (PA) signals in the corneal region. In addition, the PA signal intensity of PMTZ was slightly higher than that of MTZ. At the 12-hour time point, PMTZ still maintained a strong PA signal, verifying its superior drug retention capacity. Furthermore, the PMTZ-treated group demonstrated effective accumulation within the CNV lesion while showing minimal drug retention in normal tissues, which could benefit further *in vivo* treatment. In further studies, the *in vivo* therapeutic efficacy of PMTZ and MTZ was evaluated. Compared with the control group, both the PMTZ- and MTZ-treated groups exhibited inhibition of new blood vessel formation (Fig. 3C). Notably, the PMTZ-treated group demonstrated a more pronounced inhibitory effect than the MTZ-treated group, with most large blood vessels effectively eliminated. As CD31 is a highly specific marker for vascular endothelial cells and widely used to assess microvascular density in tissue samples, all rats were euthanized at the end of the 5-day treatment period, and the neovascularized corneas were harvested for CD31 immunohistochemical staining to evaluate angiogenesis inhibition. As shown in Fig. 3D, the PMTZ-treated group exhibited the lowest CD31 signal intensity, further confirming its superior efficacy in inhibiting CNV compared to other groups. As VEGFA and NRF2 play critical roles in angiogenesis, the expression of VEGFA and NRF2 after different treatments was detected using western blot assays. As shown in Fig. 3E–G, the protein expression level of VEGFA and NRF2 in the MTZ and PMTZ treated groups conspicuously decreases compared with the control groups.

## 4 Conclusions

In this study, we successfully engineered a novel PMTZ nanozyme with exceptional antioxidant activity. *In vitro* assays verified that PMTZ exerts potent ROS-scavenging effects under oxidative stress conditions, while cellular uptake experiments confirmed its preferential accumulation in endothelial cells of neovascular tissues. Further *in vivo* evaluations demonstrated that PMTZ nanomedicine effectively attenuates CNV, underscoring its therapeutic potential for this vision-threatening disorder. While platinum nanoparticles and Zr-based MOF carriers have been well-documented to exhibit favorable biocompatibility and negligible cytotoxicity across a wide range of *in vitro* and *in vivo* preclinical studies,^25,26^ the long-term *in vivo* biological fate of these nanomaterials in posterior/segmental ocular tissues over extended periods has not been assessed. Future studies will prioritize systematic, full-coverage pharmacokinetic and toxicokinetic analyses to bridge the existing gap between promising preclinical efficacy and real-world clinical translation. Collectively, these findings establish the PMTZ nanozyme as a promising therapeutic platform for CNV by targeting oxidative stress, thereby offering a novel strategy for clinical intervention against corneal blindness.

## Author contributions

The manuscript was written through contributions of all authors. All authors have given approval to the final version of the manuscript.

## Conflicts of interest

There are no conflicts to declare.

## Data Availability

The data that support the findings of this study are available from the corresponding author upon reasonable request
